# Connecting for health: qualitative exploration of community nursing in social prescribing for social determinants of health

**DOI:** 10.3389/fpubh.2026.1769740

**Published:** 2026-05-11

**Authors:** Ting Huang, Lei Gao, Rashid Menhas

**Affiliations:** 1 The Third People’s Hospital of Ganzhou City, Ganzhou, Jiangxi, China; 2 School of Nursing, Yingtan Health Vocational College, Yingtan, China; 3 School of Nursing, Shandong Xiehe University, Jinan, China

**Keywords:** China, community nurses, community-based care, qualitative research, social determinants of health, social prescribing

## Abstract

**Background:**

The growing recognition of social determinants of health (SDOH) has prompted health systems to adopt integrated community-based approaches beyond traditional biomedical care. Social prescribing, which connects patients to non-clinical community resources, is an emerging strategy for improving population health and supporting preventive care. In China, rapid population aging and an increasing chronic disease burden have accelerated policy interest in community-oriented healthcare models, aligned with the Healthy China 2030 initiative. Despite this shift, empirical evidence on the role of community nurses in social prescribing remains limited.

**Objective:**

This study aimed to explore community nurses contributions to the implementation of social prescribing in China and identify the key roles and processes through which they address the social determinants of health in community settings.

**Methods:**

A qualitative study was conducted in Yiwu City, Zhejiang Province, China based on constructivist grounded theory principles. Twenty-four community nurses with experience in social prescribing or related coordination practices were recruited using a theoretical sampling method for this study. Data were collected through in-depth, semi-structured interviews. Data were analyzed using Braun and Clarke’s reflexive thematic analysis to identify patterns of meaning and role development.

**Results:**

Six interconnected themes characterized community nurses’ contributions to social prescribing: (1) patient education and empowerment to enhance self-management, (2) community partnership building and resource brokerage, (3) care coordination and navigation across health and social systems, (4) strengthening social connections to reduce isolation, (5) professional role expansion and identity transformation, and (6) policy advocacy aimed at improving system integration and sustainability. The findings demonstrate that nurses function as boundary-spanning agents linking clinical services with community assets, thereby facilitating holistic, person-centered care.

**Conclusion:**

Community nurses play a central leadership role in operationalizing social prescribing within China’s community healthcare reforms. Their expanded functions support prevention, social integration, and health equity by directly addressing SDOH. Embedding nurse-led social prescribing into policy frameworks, workforce training, and integrated referral systems may strengthen sustainable community health strategies and advance healthy aging in China.

## 1 Introduction

The global health sector is undergoing a major shift, characterized by an increasing focus on tackling the social determinants of health through innovative and unconventional care models (1). This shift represents a fundamental reconceptualization of what it means to deliver effective healthcare, moving beyond the conventional boundaries of clinics and hospitals to engage with the communities and environments where people live their daily lives (2). Policymakers, healthcare administrators, and frontline practitioners increasingly recognize that medical interventions alone cannot fully account for or resolve the complex health challenges facing populations worldwide. However, there is mounting evidence that factors such as housing quality, food security, social support networks, and economic opportunities exert a profound influence on both individual and population health outcomes (3).

In China, these issues are particularly pressing due to unprecedented demographic changes and the rising prevalence of chronic illnesses that define the nation’s health landscape. The population is aging at an extraordinary rate, largely because of decades of declining birth rates and a continuous increase in life expectancy spurred by economic growth and better living conditions (4). By 2050, it is anticipated that there will be 400 million individuals over 65 years and 150 million over 80 years, a trend that will exert significant pressure on the healthcare system, social services, and family networks that have traditionally cared for older adults. This demographic transformation coincides with an epidemiological transition in which non-communicable diseases, such as cardiovascular conditions, diabetes, respiratory disorders, and mental health challenges, have replaced infectious diseases as the primary causes of morbidity and mortality (4). These conditions often necessitate ongoing management rather than immediate treatment, requiring continuous interaction between patients and their environment. To address this, the Healthy China 2030 initiative was launched as an innovative approach, emphasizing disease prevention and sustainable health management as core elements of the national health policy (5, 6). This represents the first organized attempt to connect clinical care with community-based initiatives, establishing a service network that directly addresses the socioeconomic factors affecting health while also aiming to alleviate the pressure on overburdened hospital systems (5).

Community nurses, with their holistic approach, focus on health promotion, and trusted relationships built over time with individuals from diverse populations, are ideally positioned to lead such initiatives, serving as both clinical experts and community connectors (7). However, despite the increasing recognition of the potential value of social prescribing among policymakers and international health organizations, there has been limited scholarly attention to the specific contributions of community nurses, particularly within the Chinese context, where healthcare delivery is undergoing rapid transformation amid broader social and economic changes (8).

**Figure 1:**
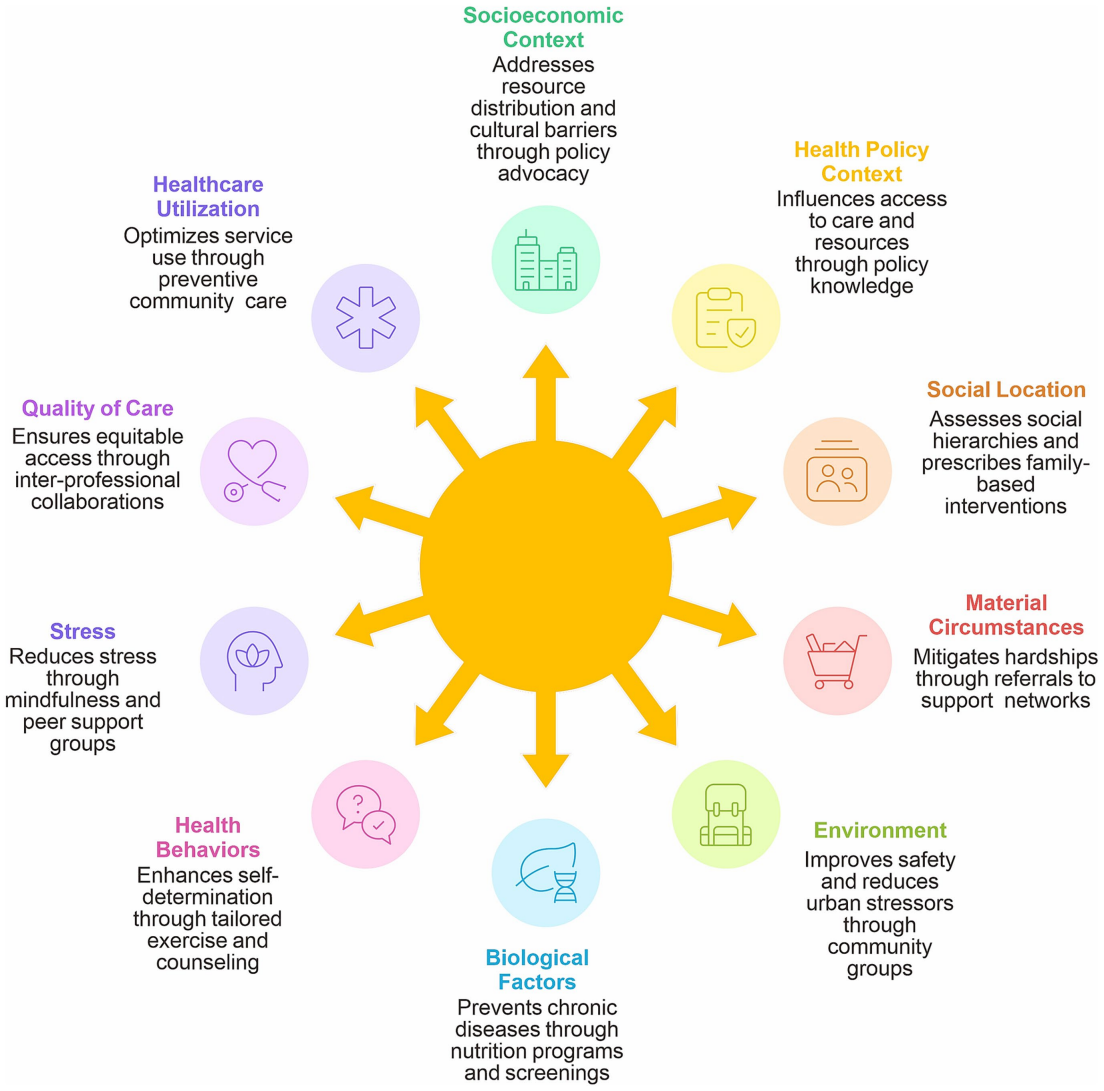
Conceptual framework.

The rationale for social prescribing is rooted in the understanding that disease is not merely a biological event but is also socially constructed through complex interactions between individuals and their environments over their life course. Structural conditions, such as income inequality, educational disparities, housing instability, employment insecurity, gender dynamics, and systemic racism, shape individuals’ exposure to health risks, influence how symptoms are interpreted and communicated, and profoundly affect the efficacy of clinical interventions when they are finally accessed (1). These social determinants are increasingly understood as the root causes of health inequities, generating systematic differences in health outcomes between population groups that cannot be explained by biological factors or healthcare access. This understanding prompts a call for interventions that target the determinants themselves rather than merely addressing their biomedical consequences through pharmaceutical or surgical means, which leave the underlying social conditions unchanged (9). Thus, social prescribing represents a practical application of social medicine principles in clinical settings, translating theoretical insights regarding the social production of health and illness into actionable patient care strategies. It encompasses a diverse range of contemporary community nursing practices, including systematic risk scoring to identify vulnerable individuals, regular home visits that provide windows into patients’ living conditions, motivational interviewing techniques that respect patient autonomy while encouraging behavior change, subtle behavioral “nudging” informed by insights from behavioral economics, and rigorous audits of referral rates to ensure equitable access to community resources (See Figure 1). These practices collectively represent a significant expansion of the nursing role beyond traditional clinical boundaries, requiring new skills, knowledge, and collaboration. This study addresses this critical gap by investigating the multifaceted roles of community nurses in implementing social prescribing in China, a context that has received minimal attention despite its significance. By giving voice to community nurses and documenting their experiences, challenges, and innovations, this study contributes to both the theoretical understanding of social prescribing implementation and practical guidance for healthcare administrators and policymakers seeking to strengthen community-based health services.

## 2 Review of literature

Globally, social prescribing has experienced significant expansion, with documented adoption in at least 17 countries across Europe, Asia, Australia and North America. Each of these regions has adapted the social prescribing model to align with their unique cultural contexts, healthcare infrastructure, and community needs (10). This adaptability is reflected in the diversity of implementation strategies, ranging from formalized referral pathways embedded within national or regional health systems to informal grassroots initiatives driven by local organizations and community groups. These variations demonstrate the flexibility of social prescribing as a framework capable of responding to different healthcare environments and population requirements. The broad uptake of social prescribing underscores a paradigm shift in healthcare delivery, in which social determinants are no longer peripheral considerations but are integral components of patient care. This transition reflects the growing awareness that health outcomes are deeply influenced by factors outside the clinical setting and that addressing these factors can lead to more holistic, effective and sustainable health interventions. By incorporating social prescribing into mainstream healthcare strategies, systems are moving toward a more comprehensive model that prioritizes patient-centered care and recognizes the value of community resources in supporting health and well-being (10).

### 2.1 Social prescribing as care integration strategy

Social prescribing has evolved from grassroots community development initiatives into formalized healthcare interventions across multiple national contexts (11). The United Kingdom’s National Health Service has been at the forefront of this evolution, establishing link worker roles and developing standardized referral pathways (12). Evaluation research has demonstrated positive effects on psychological well-being, social connectedness, and healthcare utilization, although effect sizes vary across populations and implementation contexts (13). Implementation research has identified several factors influencing the effectiveness of social prescribing, including the quality of relationships between referrers and link workers, the availability and accessibility of community resources, and the degree of integration between clinical and community systems (14). It examines how frontline practitioners, particularly nurses, negotiate the practical challenges of implementing social prescriptions within the existing care workforce in the UK. This gap is particularly pronounced in contexts where social prescribing is still emerging and the formal infrastructure remains underdeveloped. Social prescribing is a transformative approach within healthcare that shifts the focus from purely medical interventions to addressing the broader social determinants of health. This approach has been increasingly institutionalized, particularly in the UK, where the National Health Service (NHS) has formalized roles, such as link workers, who act as intermediaries to connect patients with community-based resources (15). These link workers facilitate standardized referral pathways to ensure that patients receive tailored social support and clinical care in the community. Evidence from evaluation studies underscores the benefits of social prescribing, highlighting improvements in psychological well-being and social connectedness, and potential reductions in healthcare utilization. However, the magnitude of these effects is not uniform and varies depending on demographic factors, local implementation strategies, and the maturity of social prescribing infrastructure (16).

### 2.2 Social prescribing perspective in China

Social prescribing, as perceived in China, represents a nascent yet promising paradigm shift toward integrating non-clinically based community interventions into healthcare to enhance overall well-being, particularly amid rapid population aging in China. Conceptually introduced in 2018 through academic discourse inspired by UK models, it is not yet fully institutionalized but is increasingly aligned with national health strategies, such as the Healthy China 2030 blueprint, which prioritizes prevention and the social determinants of health (17). Chinese scholars and practitioners view it as a holistic mechanism that extends beyond symptom treatment to address root causes, such as social isolation, economic pressures, and environmental factors, often through referrals to local activities, such as group hobbies, volunteering, or educational sessions (18). This perspective underscores the importance of cultural adaptation, where collective community involvement supersedes individualistic approaches, with multidisciplinary teams rather than solo link workers facilitating assessments and connections during primary care visits or annual health check-ups (19). Pilot initiatives, notably in regions such as Shangrao (Jiangxi Province), have piloted this model under the guidance of the World Health Organization (WHO), focusing on the mental health of older adults and demonstrating improvements in cognitive function and social participation through pre- and post-intervention evaluations (18). A distinctive Chinese lens on social prescribing integrates Traditional Chinese Medicine (TCM) to enhance cultural relevance and accessibility, viewing it as a bridge between ancient preventive principles and modern community care (17). In terms of institutional mechanisms, social prescribing in China operates through a multilevel initiation process. Unlike the UK’s formalized link worker model, where general practitioners (GPs) typically initiate referrals, China’s approach involves multiple entry points: community nurses identify needs during routine home visits and health screenings, primary care physicians refer patients during clinical consultations, and community health center administrators coordinate with local government health initiatives. Referrals are structured through the existing community health service network, with community nurses serving as primary coordinators rather than specialized link workers. Community nurses hold significant authority in assessing social needs, mapping local resources, and facilitating connections; however, their role differs from that of UK link workers in that they maintain concurrent clinical responsibilities. This model represents a hybrid approach that integrates social prescribing functions within existing community nursing roles, rather than creating distinct positions. Compared to international examples, particularly the UK’s National Health Service model with dedicated link workers and standardized pathways, China’s approach is more decentralized and embedded within the broader community health workforce, reflecting the country’s integrated primary healthcare system and the prominent role of community nurses as frontline health providers (17, 19).

### 2.3 Chinese community nursing scenario and social healthcare

Social healthcare in China at the community level adopts a social prescription approach, connecting individuals to non-clinical resources such as cultural activities, nature-based exercises, and digital tools to promote healthy aging and address the social determinants of health (19). The concept of person-centered care has gained traction in Chinese nursing scholarship, emphasizing holistic assessment, shared decision-making, and attention to patient preferences and values. Implementing person-centered approaches within social prescribing contexts requires nurses to engage with patients’ social circumstances, cultural backgrounds, and community connections. This represents a significant expansion of traditional biomedical care models and requires new competencies and organizational support. China’s community nursing and social healthcare systems have undergone significant transformations since the 2009 comprehensive primary healthcare (PHC) reforms, which aimed to shift from a hospital-centric model to one that emphasizes accessible, equitable, and community-based services (20). Community nursing, with its roots in the integration of nursing and public health over the past three decades, now involves approximately 250,000 registered nurses delivering clinical interventions, chronic disease management, and older adults care across nearly 800 urban facilities. Nurses act as “agents of the state” in health promotion, using biopower techniques such as surveillance follow-ups and data collection to regulate behaviors, although patient noncompliance and privacy concerns persist (21, 22). Within the social prescribing context, community nurses exercise considerable autonomy in identifying patients with social needs, assessing community resources, and referring them to non-clinical services. Their authority stems from their position as trusted healthcare providers with established patient relationships rather than from formal link worker designations. This embedded authority enables nurses to effectively bridge the clinical and community sectors, although it also means that social prescribing responsibilities are added to existing heavy workloads. Expectations for improvement involve broader coverage (e.g., fall risk assessments), diversified training (e.g., simulations), and policy support for insurance inclusion and clear practice boundaries (23).

### 2.4 Theoretical framework

The theoretical framework for this qualitative study was based on the Dahlgren-Whitehead model. Health outcomes are profoundly influenced by social determinants of health (SDOH), which encompass structural factors such as socioeconomic status, education, employment, social support networks, and environmental conditions. The Dahlgren-Whitehead model provides a foundational structure for understanding these determinants as layered influences, ranging from individual lifestyle factors to broader socioeconomic and political contexts (53). This ecological perspective highlights the interplay between the individual, community, and societal levels, emphasizing that addressing SDOH requires interventions beyond clinical care to promote health equity and improve health outcomes. Social prescribing has emerged as a key mechanism for addressing the SDOH by connecting individuals to nonclinical community resources and activities that enhance well-being, social connectedness, and self-management of health. Globally, social prescribing has evolved as an integrative approach that links healthcare systems to community assets, particularly in primary and community care settings (10, 24). Self-determination theory further illuminates this process, positing that effective social prescribing fulfills basic psychological needs for autonomy, competence, and relatedness, thereby fostering intrinsic motivation, empowerment, and sustained engagement in health-promoting activities (25). In community nursing, this framework positions nurses as pivotal facilitators of social prescribing initiatives. Community nurses, with their proximity to patients and understanding of local resources, play a multifaceted role in identifying SDOH-related needs, co-designing personalized referrals, and bridging clinical and community sectors to mitigate health inequities (26). In China, social prescribing aligns with national priorities, such as the Healthy China 2030 initiative, where community-based practices, including health education, cultural activities, nature-based interventions, and digital support, target healthy aging and reduce reliance on medical services by leveraging non-profit and local organizational networks (19). Thus, this study draws on these integrated theoretical elements to explore the contributions of community nurses in China’s social prescribing landscape and identify themes that define their role in addressing the SDOH.

## 3 Methods

This study employed a constructivist grounded theory methodology that emphasizes the co-construction of knowledge between researchers and participants while maintaining systematic analytical procedures (27). This approach was selected for its capacity to generate theoretical explanations of social processes from the perspectives of those engaged in them (28). Focusing on how community nurses negotiate boundary-spanning roles aligns well with the emphasis on process and action in grounded theory (29). The study was conducted in accordance with the ethical principles outlined in the Declaration of Helsinki and was approved (K2023034) by the Ethics Committee of the Fourth Affiliated Hospital of Zhejiang University, Yiwu, China. All participants provided written informed consent after receiving detailed information about the study’s purpose, procedures, and confidentiality. Pseudonyms were used in reporting findings to protect the participants’ anonymity.

### 3.1 Study setting and participants

The study was conducted in Yiwu City, Zhejiang Province, a rapidly urbanizing region in eastern China with a population of approximately 1.9 million. Yiwu was selected as the research site because of its active implementation of community-based healthcare reforms and ongoing social-prescribing pilot initiatives. The city’s mix of urban and rural healthcare settings provided opportunities to examine the boundaries of diverse implementation contexts.

Participants were recruited using theoretical sampling, a grounded theory technique in which sample selection is guided by emerging analytical categories (30). Theoretical sampling, a core grounded theory technique, guided the iterative selection of participants to ensure diverse perspectives and saturation of emerging analytical categories. However, for data analysis, we employed Braun and Clarke’s reflexive thematic analysis, which provided a systematic framework for identifying patterns of meaning across the dataset. This integration allowed us to leverage the sampling rigor of grounded theory while utilizing the structured approach of thematic analysis to theme development, which is particularly suited for exploring professional roles and experiences. Initial participants were identified through professional networks and snowball sampling, with subsequent participants selected to fill emerging theoretical categories and explore variations in practice contexts (31). The inclusion criteria required participants to be registered nurses working in community healthcare settings with direct experience in social prescribing or related care coordination.

#### 3.1.1 Data collection tool

The interview guide was developed based on a preliminary review of the literature and refined through pilot interviews with two community nurses not included in the main study [54]. Open-ended questions explored participants’ experiences with social prescribing implementation, including how they identified patients’ social needs, connected patients with community resources, navigated interprofessional relationships, and negotiated role boundaries.

#### 3.1.2 Data collection

Data were collected through in-depth semi-structured interviews conducted between April and August 2023. The first author, trained in qualitative research methods and with prior experience in Chinese healthcare settings, conducted the interviews in Mandarin Chinese. The interviews lasted between 60 and 120 min (mean = 78 min) and were conducted in private locations convenient for the participants to ensure confidentiality. Specifically, initial interviews revealed variations in nursing practice across urban, suburban, and rural settings, prompting deliberate recruitment from each context. As themes related to professional role expansion emerged, we purposively sought nurses with varying levels of social prescribing experience (less than 1 year, 1–2 years, and more than 2 years) to ensure comprehensive coverage of the phenomenon. This iterative sampling process (28, 32) continued until thematic saturation was achieved, with no new substantive themes emerging from additional interviews. Field notes were maintained after each interview to document contextual observations, researcher reflections, and emerging analytical insights. These notes contributed to the analytical process by providing additional context for interpreting the interview data and supporting the reflexive examination of the researcher’s assumptions and their positionality.

#### 3.1.3 Data analysis

The transcripts were analyzed thematically using Braun and Clarke’s six-phase approach to thematic analysis. Thematic analysis is a method for systematically identifying, organizing, and offering insights into patterns of meaning (themes) in a qualitative dataset. This allows researchers to understand the shared meanings and experiences of the data. Unlike more prescriptive methods such as grounded theory or interpretative phenomenological analysis, TA is theoretically flexible and can be applied within realist/essentialist, contextual, or constructivist frameworks (33). This approach was chosen for its theoretical flexibility and systematic procedure for identifying, analyzing, and reporting patterns in qualitative data. While theoretical sampling guided data collection to ensure comprehensive coverage of the phenomenon, the six-phase thematic analysis process (familiarization, coding, theme generation, theme review, theme definition, and report writing) provided a structured analytical framework. The integration of these approaches ensured that theme development was both data-driven (through theoretical sampling) and systematically organized (through a reflexive thematic analysis).

### 3.2 The reflexive approach and process

In the reflexive TA approach developed by Braun and Clarke, the researcher plays an active and subjective role in interpreting the data, rather than aiming for coder reliability or consensus. The process typically follows six recursive phases: (1) familiarizing oneself with the data, (2) generating initial codes, (3) constructing themes, (4) reviewing potential themes, (5) defining and naming themes, and (6) producing the report (52). This method prioritizes depth, richness, and organic theme development over rigid theme development.

## 4 Results

### 4.1 Demographic profile of the study participants

The study included 24 participants, the majority of whom practiced in urban community health centers (12, 50.0%), followed by those in suburban clinics (7, 29.2%) and rural health stations (5, 20.8%). The sample was predominantly female (22, 91.7%), with only two male participants (8.3%). Regarding years of experience, the largest group had 6–10 years (6, 27.3%), while those with 11–15 years accounted for 5 participants (22.7%), 16–20 years for 4 (18.2%), and 21 + years for 3 (13.6%); data for this variable were reported for 22 participants. In terms of education, over half held a bachelor’s degree (12, 54.5%), eight held an Associate Degree (36.4%), and two held a master’s degree (9.1%). Finally, regarding social prescribing experience, 10 participants (45.5%) had 1–2 years of experience, while 6 (27.3%) had less than 1 year, and another 6 (27.3%) had more than 2 years (Table 1).

**Table 1:** Demographics of the study participants (*N =* 24).

Demographic variable	Categories	Frequency/percentage
Practice Setting	Urban Community Health Center	12 (50.0%)
	Suburban Clinic	7 (29.2%)
	Rural Health Station	5 (20.8%)
Gender	Male	2 (8.3%)
Years of Experience	Female	22 (91.7%)
	6–10 years	6 (27.3%)
	11–15 years	5 (22.7%)
	16–20 years	4 (18.2%)
	21 + years	3 (13.6%)
Education Level	Associate Degree	8 (36.4%)
	Bachelor’s Degree	12 (54.5%)
	Master’s Degree	2 (9.1%)
Social Prescribing Experience	Less than 1 year	6 (27.3%)
	1–2 years	10 (45.5%)
	More than 2 years	6 (27.3%)

#### 4.1.1 Major extracted themes

The thematic analysis table (Table 2) presents six key themes identified from semi-structured interviews with nurses, analyzed using Braun and Clarke’s thematic analysis approach. Theme T1, Patient Education and Skill Empowerment, emphasizes equipping patients with knowledge and practical skills to promote self-management and autonomy. Theme T2, Community Partnerships and Resource Connections, highlights the role of forging collaborations with community organizations to access vital resources. Theme T3, Care Coordination and System Navigation, positions nurses as central coordinators who navigate patients through intricate health and social service systems. Theme T4, Social Connection and Support Building, focuses on combating social isolation by cultivating networks and enhancing social capital. Theme T5, Professional Role Development and Identity, addressed the cultivation of advanced competencies and the expansion of nursing roles. Finally, Theme T6, Policy Advocacy and Systemic Improvement, underscores efforts to advocate for policy reforms and broader systemic enhancements in healthcare delivery.

**Table 2:** Main extracted themes.

Theme code	Theme title	Theme description
T1	Patient Education and Skill Empowerment	Equipping patients with knowledge and practical skills to enhance self-management and autonomy is essential.
T2	Community Partnerships and Resource Connection	Forging collaborations with community organizations to identify and secure essential resources is crucial.
T3	Care Coordination and Community System Navigation	Positioning nurses as key coordinators who guide patients through complex health and social service systems is essential.
T4	Social Connection and Support Building	Addressing social isolation by fostering networks and strengthening social capital.
T5	Professional Role Development and Identity	Cultivating advanced competencies and expanding the scope of nursing practices.
T6	Policy Advocacy and Systemic Improvement	Promoting policy reforms and broader systemic changes to improve healthcare delivery is essential.

#### 4.1.2 Theme explanations

##### 4.1.2.1 (T1) patient education and skill empowerment

The first theme centered on the expanded role of community nurses as educators and facilitators of patient empowerment through tailored health education and skill building. Nurses described moving beyond traditional medical instruction to a broader conceptualization of health literacy that encompassed practical life skills, self-advocation, and confident navigation of healthcare and community systems.

*They emphasized enabling patients to take ownership of their health and social well-being, recognizing that effective self-management often depends on non-clinical competencies. “We want patients to gain the confidence and tools to speak up for their needs and manage their conditions independently, without always needing us to intervene” (CN 3, 8). “It’s not just about telling them what to do; it’s teaching them how to understand their options and make informed choices that fit their lives” (CN 1, 6). “Many patients feel overwhelmed by information—we break it down, use simple language, and practice skills together until they feel capable” (CN 4, 10).*

Nurses reported adapting educational approaches to individual learning styles, cultural backgrounds, and digital literacy levels by incorporating interactive methods, such as teach-back techniques, visual aids, goal-setting worksheets, and digital health tutorials. Programs frequently included one-on-one coaching, group workshops, and follow-up sessions covering chronic disease management, medication adherence, communication skills for appointments, financial literacy for accessing services, nutrition planning, and the use of health apps and wearable devices.

##### 4.1.2.2 (T2) community partnerships and resource connection

The second theme focused on nurses’ function as resource brokers who forge and maintain partnerships with community organizations to link patients with non-clinical support services. Participants viewed social prescribing as a core mechanism for addressing the social determinants of health, deliberately connecting patients to local resources that promote well-being beyond medical treatment alone.

*They highlighted the importance of building trusted and reciprocal relationships with community entities to ensure sustainable referral pathways for patients. “Our role is to know the community inside out and act as the bridge that gets patients to the right support at the right time” (CN 2, 5, 9).*

*“We spend time nurturing these partnerships. It is not just a directory of services; it is real relationships that make referrals work” (CN 1, 7).*

*“Patients often do not know what is available locally. We map it out and connect them personally so they feel supported from the start” (CN 4, 10).*

Nurses described actively mapping local assets, such as food banks, exercise groups, arts programs, volunteering services, and debt advice centers, and developing formal and informal collaboration protocols with community partners to address the SDOH. Referrals were personalized, with follow-up to confirm engagement, and nurses often co-facilitated introductory sessions, provided transport assistance, or accompanied patients to initial appointments to reduce barriers to service uptake.

##### 4.1.2.3 (T3) care coordination and system navigation

The third theme positioned community nurses as central coordinators and navigators, guiding patients through fragmented health and social care systems. Participants emphasized their unique overview of patients’ needs, enabling them to orchestrate multidisciplinary support and prevent care gaps in the future. Nurses viewed themselves as pivotal in translating complex system requirements into actionable steps for patients seeking multiple services.

*“Patients often feel lost in the system; we become their guide, making sure nothing falls through the cracks” (CN 1, 4, 7).*

*“Coordination is about seeing the whole picture and pulling all the threads together so the patient doesn’t have to” (CN 3, 8).*

*“We’re the constant presence when services change or overlap, we step in to advocate and keep everything on track” (CN 6, 9).*

They reported proactive case management, including scheduling appointments across providers, liaising with social workers, housing services, benefits advisors, advocating during multidisciplinary meetings, and maintaining shared care plans. Particular attention was given to vulnerable populations with complex needs, where nurses facilitated transitions between hospital and community settings, ensured continuity during changes in social circumstances, and supported the application of disability aids and home adaptations.

##### 4.1.2.4 (T4) social connection and support building

The fourth theme addressed nurses’ deliberate efforts to combat social isolation by fostering social connections and strengthening patients’ social capital. Participants recognized loneliness and limited social networks as significant barriers to health, positioning social prescribing as a key strategy for building supportive relationships and integrating into the community. They stressed the therapeutic value of meaningful social contact and a sense of belonging.

*“Many of our patients are isolated; connecting them to groups or activities can be as impactful as any medication” (CN 6, 10).*

*“We see the difference a friendly group makes patients light up when they feel part of something again” (CN 2, 5).*

*“It’s about rebuilding confidence step by step; some need encouragement just to attend once, but then they thrive” (CN 1, 8).*

Nurses facilitated referrals to befriending services, peer support groups, community centers, volunteering opportunities, interest-based clubs, and walking groups, and organized nurse-led social events such as coffee mornings or gardening sessions. They monitored engagement and provided ongoing encouragement, often helping patients overcome initial anxiety about participation, addressing practical barriers such as transportation, and celebrating small successes in relationship building.

##### 4.1.2.5 (T5) professional role development and identity

The fifth theme explored the evolution of professional identity as community nurses embraced expanded roles within the social-prescribing framework. Participants described a shift from traditional clinical tasks toward holistic, preventive, and relational practices that require new competencies in community engagement, advocacy, and interdisciplinary collaboration. This expansion was viewed as both challenging and professionally rewarding, affirming nurses’ value in addressing broader health determinants.

*“Social prescribing has transformed what it means to be a community nurse it’s given us scope to use our skills in ways that truly make a difference” (CN 2, 5, 8).*

*“It’s stretched us professionally, but in a good way we’re more than clinical experts now; we’re enablers of wider change” (CN 3, 7).*

*“Developing this role has built my confidence; I feel empowered to lead and innovate in patient care” (CN 4, 9).*

Nurses pursued targeted training in motivational interviewing, cultural competence, asset-based community development, trauma-informed care, and evaluation of non-clinical outcomes. They reflected growing confidence in autonomous decision-making, leadership in multidisciplinary teams, boundary spanning with non-health sectors, and the development of a distinct professional identity centered on empowerment, prevention, and social justice.

##### 4.1.2.6 (T6) policy advocacy and systemic improvement

The sixth theme captured nurses’ commitment to policy advocacy and driving systemic change to embed social prescribing in the mainstream healthcare. Participants identified structural barriers, such as funding limitations, fragmented services, and the undervaluation of non-clinical interventions, and positioned themselves as informed advocates for reform in mental health services. They sought to influence decision-makers by sharing frontline evidence of their impact.

*“We see the gaps every day; it’s our responsibility to push for policies that make social prescribing accessible to everyone who needs it” (CN 3, 7, 9).*

*“Advocacy means taking our stories and data to those who can change things we cannot just stay at the bedside” (CN 1, 6).*

*“If we don’t speak up for sustainable funding and better integration, these valuable approaches will remain on the fringes” (CN 5, 10).*

Nurses engaged in activities, including contributing to local health strategy consultations, presenting outcome data to commissioners and integrated care boards, collaborating on pilot evaluations and research studies, and networking with professional bodies to promote the recognition of expanded nursing roles. They advocated for sustainable funding models, integrated digital referral systems, standardized training pathways, and workforce development to support the widespread adoption and equity of social-prescribing practices.

## 5 Discussion

The thematic analysis conducted in this study illuminates the multifaceted and expanding roles of community nurses in the implementation of social prescribing, a person-centered approach that connects individuals to non-clinical community resources to address social determinants of health and enhance overall well-being. These findings contribute to the growing body of evidence on social prescribing as an innovative strategy to complement traditional medical care, particularly in community settings where nurses maintain longitudinal relationships with patients (10, 34, 35). The identified themes reflect a paradigm shift toward holistic, preventive, and relational nursing practice, aligning with international developments that emphasize the integration of social interventions to reduce healthcare utilization and improve outcomes for chronic conditions and social isolation (36–38).

Theme T1, Patient Education and Skill Empowerment, underscores community nurses’ role in delivering tailored education to foster self-management, health literacy, and autonomy. By incorporating interactive methods and addressing cultural and digital competencies, nurses can enable patients to navigate health and social systems independently of them. This aligns with evidence indicating that social-prescribing interventions enhance the self-management of long-term conditions through skill-building and empowerment strategies (39, 40). Similar nurse-led approaches have demonstrated improvements in patient confidence and quality of life, particularly for chronic diseases such as diabetes and cancer (41, 42).

The themes of community partnerships and resource connections (T2) and Care Coordination and System Navigation (T3) highlight nurses’ brokerage and navigational functions, which involve asset mapping, personalized referrals, and multidisciplinary coordination. These roles facilitate access to local resources and prevent care fragmentation, particularly among vulnerable groups. Such practices are consistent with global models in which link workers or navigators bridge clinical and community services, yielding sustainable pathways and reducing barriers to engagement (37, 43, 44). Comparative analyses across high-income countries further support the value of interprofessional partnerships in embedding social prescriptions in primary and community care (38, 45).

Theme T4, Social Connection and Support Building, emphasizes nurses’ efforts to mitigate loneliness and isolation through referrals to social activities and peer networks, recognizing their equivalent therapeutic impact on clinical interventions. This finding is corroborated by systematic reviews demonstrating the positive effects of social prescribing on social capital, belonging, and mental well-being, particularly among older adults and those with long-term conditions (44, 46, 47). Interventions targeting loneliness via community connections have shown reductions in isolation and enhancements in psychosocial outcomes, underscoring the relevance of community nursing in aging populations (34).

The themes of Professional Role Development and Identity (T5) and Policy Advocacy and Systemic Improvement (T6) illustrate the transformative influence of social prescribing on nursing practice, involving the acquisition of new competencies in advocacy and collaboration, alongside efforts to address structural barriers. Nurses’ advocacy for funding, integration, and recognition reflects frontline insights into implementation challenges (35, 43). Economic evaluations highlight positive returns on investment for social prescribing, supporting calls for policy reforms to sustain expanded roles (48). Interprofessional collaboration is essential for overcoming role ambiguities and fostering person-centered delivery (45, 49).

Collectively, these themes affirm the strategic positioning of community nurses in leading social prescribing initiatives, leveraging their community embeddedness and holistic expertise (35, 42). Practical implications include targeted training in asset-based and motivational approaches, while policy recommendations advocate integrated systems and equitable funding (10, 34, 50, 51). The strengths of this study include the rich qualitative data from practicing nurses, which offers practical insights into implementation. The limitations of this study include context-specific findings and potential transferability constraints. Future research should prioritize longitudinal evaluations and cross-cultural comparisons to strengthen the evidence base (39, 40).

## 6 Conclusion

This thematic analysis illuminates the pivotal and multifaceted roles of community nurses in advancing social prescribing, revealing their essential contributions as educators empowering patient autonomy, brokers forging community partnerships, coordinators navigating complex systems, connectors fostering social capital, innovators evolving professional identities, and advocates driving systemic reform. These findings underscore the transformative potential of social prescribing in addressing the social determinants of health, mitigating isolation, and enhancing holistic well-being, particularly among individuals with chronic conditions in community settings. By leveraging their embedded position and relational expertise, community nurses are ideally suited to lead these initiatives, aligning with global evidence of the efficacy of nonclinical interventions in reducing healthcare burdens and improving outcomes. This study highlights critical implications for nursing practice, including the need for enhanced training in asset-based approaches and advocacy skills, as well as policy imperatives for sustainable funding, integrated referral systems, and workforce recognition to embed social prescribing into healthcare frameworks. Ultimately, embracing these expanded roles enriches nursing practice and advances a more preventive, person-centered paradigm in healthcare delivery, offering profound opportunities to foster healthier communities and reduce disparities in access to support services.

### 6.1 Implications

The findings of this study have significant implications for nursing practice, healthcare policy, and education in the context of social prescription. For nursing practice, the identified themes highlight the need to formally recognize and support the expanded roles of community nurses as educators, resource brokers, coordinators, social connectors, role innovators, and policy advocates. Healthcare organizations should prioritize professional development programs that equip nurses with advanced skills in motivational interviewing, asset-based community development, cultural competence, and digital navigation tools to enhance their effectiveness in delivering personalized and holistic interventions. Furthermore, integrating social prescribing into routine community nursing workflows could optimize patient outcomes by systematically addressing the social determinants of health, reducing the reliance on clinical services, and promoting preventive care. At the policy level, this study underscores the necessity of sustained funding models, integrated referral platforms, and regulatory frameworks that embed social prescriptions into primary and community care systems. Policymakers should advocate equitable resource allocation to support interprofessional collaboration and address structural barriers, such as fragmented services, to ensure accessibility for vulnerable populations. In nursing education, curricula should incorporate modules on social prescribing principles, interdisciplinary teamwork, and advocacy to prepare future nurses for these evolving roles, fostering a workforce aligned with person-centered and preventive healthcare paradigms.

### 6.2 Future research direction

Future research should build on these qualitative insights by employing longitudinal designs to evaluate the long-term impact of nurse-led social prescribing on patient outcomes, including health-related quality of life, healthcare utilization and social capital. Quantitative or mixed-methods studies are recommended to measure the efficacy and cost-effectiveness of these interventions across diverse populations and settings, facilitating generalizability beyond the current context of this study. Comparative analyses across different healthcare systems or countries would provide valuable perspectives on the contextual factors influencing the success of implementation. Additionally, investigations focusing on specific patient groups, such as older adults, individuals with mental health conditions, or ethnic minorities, could elucidate tailored approaches and address issues of equity. Exploring barriers and enablers from the perspectives of patients and community partners would complement the nurse-centered focus of this study and inform more inclusive models of care. Finally, interventional studies testing standardized training programs or digital tools for social prescribing could generate evidence to guide the scalable adoption and refinement of such policies.

### 6.3 Study limitations

This study had several limitations that warrant consideration when interpreting its findings. As a qualitative thematic analysis based on semi-structured interviews with a relatively small sample of community nurses (*n* = 24) from a specific healthcare context, the results may not be fully generalizable to broader nursing populations or diverse geographical and sociocultural settings. The reliance on self-reported data from nurses introduces potential recall bias and social desirability effects, where participants may have emphasized the positive aspects of their roles or overlooked the challenges. Furthermore, the study captures only the perspectives of community nurses, excluding insights from patients, community partners, or other healthcare professionals, which could provide a more comprehensive understanding of the implementation and outcomes of social prescribing. The cross-sectional design limited the ability to examine longitudinal changes in nursing roles or the sustained impact of the social prescribing interventions. Finally, while Braun and Clarke’s thematic analysis approach was rigorously applied, the interpretive nature of qualitative research inherently involves researcher subjectivity, which may influence theme identification and descriptions, despite efforts to ensure reflexivity and transparency. These limitations highlight the need for future studies to address these issues using larger, multi-perspective, and longitudinal methodologies.

## Data Availability

The raw data is available upon reasonable request to the corresponding author.
